# The emerging scenario of immunotherapy for T-cell Acute Lymphoblastic Leukemia: advances, challenges and future perspectives

**DOI:** 10.1186/s40164-022-00368-w

**Published:** 2023-01-09

**Authors:** Daniele Caracciolo, Antonia Mancuso, Nicoletta Polerà, Caterina Froio, Giuseppe D’Aquino, Caterina Riillo, Pierosandro Tagliaferri, Pierfrancesco Tassone

**Affiliations:** 1 Department of Experimental and Clinical Medicine, Magna Græcia University, Catanzaro, Italy; 2 Sbarro Institute for Cancer Research and Molecular Medicine, Center for Biotechnology, College of Science and Technology, Temple University, Philadelphia, PA USA

**Keywords:** T-ALL, Acute Lymphoblastic Leukemia, CAR-T, Bispecific T-cell engagers, BTCEs, Immunotherapy, Hematological malignancies

## Abstract

T-cell acute lymphoblastic leukemia (T-ALL) is a challenging pediatric and adult haematologic disease still associated with an unsatisfactory cure rate. Unlike B-ALL, the availability of novel therapeutic options to definitively improve the life expectancy for relapsed/resistant patients is poor. Indeed, the shared expression of surface targets among normal and neoplastic T-cells still limits the efficacy and may induce fratricide effects, hampering the use of innovative immunotherapeutic strategies. However, novel monoclonal antibodies, bispecific T-cell engagers (BTCEs), and chimeric antigen receptors (CAR) T-cells recently showed encouraging results and some of them are in an advanced stage of pre-clinical development or are currently under investigation in clinical trials. Here, we review this exciting scenario focusing on most relevant advances, challenges, and perspectives of the emerging landscape of immunotherapy of T-cell malignancies.

## Background

Acute lymphoblastic leukemia (ALL) is a heterogeneous disease characterized by proliferation and accumulation of immature lymphoid cells in bone marrow, peripheral blood, lymphoid tissues, and other extra-nodal sites [1]. In accordance with the definition of the World Health Organization (WHO), ALL can be classified as B or T-cell acute lymphoblastic leukemia (B- or T-ALL) [2, 3]. It occurs more frequently in males than females and in children than in adults [4]. T-ALL aetiology is probably the combination of environmental causes, such as radiation or other agents (benzene, pesticides, bioflavonoids, etc.) exposure and genetic susceptibility. Pathogenetic mechanisms include functional loss of tumor-suppressor genes, activation of oncogenes or translocation events leading to new chimeric proteins, with oncogenic potential in T cell progenitors and driving leukemic transformation. The most common genetic abnormalities of T-ALL are structural chromosomal alterations that lead to secondary somatic DNA copy number variations and mutations [5–7]. Also, specific DNA methylation patterns correlate with clinical outcome in ALL patients, suggesting that methylation analysis may be of help for classifying ALL patients’ subtypes [8]. Based on genetic lesions, T-ALL could be classified into type A and type B [9]. In particular, type A mutations involve driver oncogenes or oncogene fusions (*HOXA, MYB, TAL*/*LMO, TLX1, TLX3*), while type B mutations activate the NOTCH1 pathway in over than 60% of T-ALL cases, or, in the remaining part of the cases, activate cytokine signalling pathways (IL7R, JAK1 / 3, FLT3, CKIT, PI3K / AKT / PTEN, ABL1, N / KRAS) and factors involved in transcription (RUNX1, ETV6, BCL11B, WT1, TCF7, LEF1, CTNNB1, GATA3, IKZF1), inactivate cell cycle inhibitors (CDKN2A / B, CDKN1B, CDKN1C, CCND3, RB) or deregulate chromatin modifiers and remodelling factors (PHF6, CTCF, KDM6A, SETD2, KMT2A / 2D / 2C, DNMT3A, IDH1 / 2) [10, 11].

Based on the expression of CD1a, CD3, CD5, CD7, and TdT, different immunophenotypic subgroups have been distinguished for T-ALL: pro-T, pre-T, cortical, and mature [12]. Recently, a new provisional entity, which accounts for approximately 10% of pediatric and 40–50% of adult T-ALL cases, was introduced. The early T-cell precursor lymphoblastic leukemia (ETP-ALL) is characterized by the lack of CD1a and CD8, weak expression of CD5, and expression of stem cell (CD34, CD117), and myeloid (CD13, CD33) lineage markers. This entity has been associated with poor prognosis, albeit intensified chemotherapy regimens have improved the outcome [13].

To date, the standard front-line therapy for T-ALL is represented by intensive chemotherapy and central nervous system (CNS) prophylaxis [14]. Vincristine, prednisone, and anthracyclines, sometimes associated with L-asparaginase, are the most active drugs. Cytosine-arabinoside and high-dose methotrexate (MTX) for CNS prophylaxis are also commonly used [15, 16]. Through these approaches, the estimated 5-year EFS and OS of T-ALL patients are 83.8% and 89.5%, respectively [17]. Adult T-ALL patients still have poor outcomes and lower survival than young ones. Survival rate of pediatric patients is around 90%, while in adults it is between 30–40% [18, 19]. Moreover, relapses often occur in adult T-ALL patients (40–75% *vs* 15–20% in pediatric patient), and cure rates are less than 10% among this group of patients [20, 21]. The minimal residual disease (MRD) is the most relevant prognostic factor of relapse [22]. Nelarabine, the only new therapy recently introduced, is a nucleoside analog indicated for the treatment of pediatric and adult patients who are relapsed and/or refractory (r/r) after at least two chemotherapy regimens. However, it slightly impacts the disease progression in a substantial minority of patients (30%) [23]. Allogeneic haematopoietic cell transplantation (HCT) has a relevant role in patients with high-risk or r/r disease. Nevertheless, also in patients who respond to nelarabine and are consolidated with allogeneic stem cell transplantation (SCT), the outcome remains extremely poor [24]. Finally, γ-Secretase inhibitors hold promise for the treatment of patients with NOTCH1 mutations, and the results of clinical trials investigating these agents are eagerly awaited [25].

We here review the potential immunotherapeutic approaches for T-ALL, focusing on monoclonal antibodies (mAbs), chimeric antigen receptor (CAR) T-cells and Bispecific T-Cell Engagers (BTCEs) as promising and emerging strategies to increase cure rates and reduce the burden of intensive and prolonged maintenance chemotherapy.

## Monoclonal antibodies (mAbs) and chimeric antigen receptor (CAR) T-cells for the treatment of T-ALL

Presently, unlike B-ALL, immunotherapeutic attempts for T-ALL patients have been mostly hampered by the shared expression of surface antigens among normal cells and leukemic T cells [26–30]. Indeed, the search and identification of selective targets for T-ALL blasts not expressed by normal T cells remains the main challenge. However, new targets are presently under investigation for novel immunotherapeutic strategies based on mAbs and chimeric antigen receptor (CAR)-T cells.

**Fig. 1:**
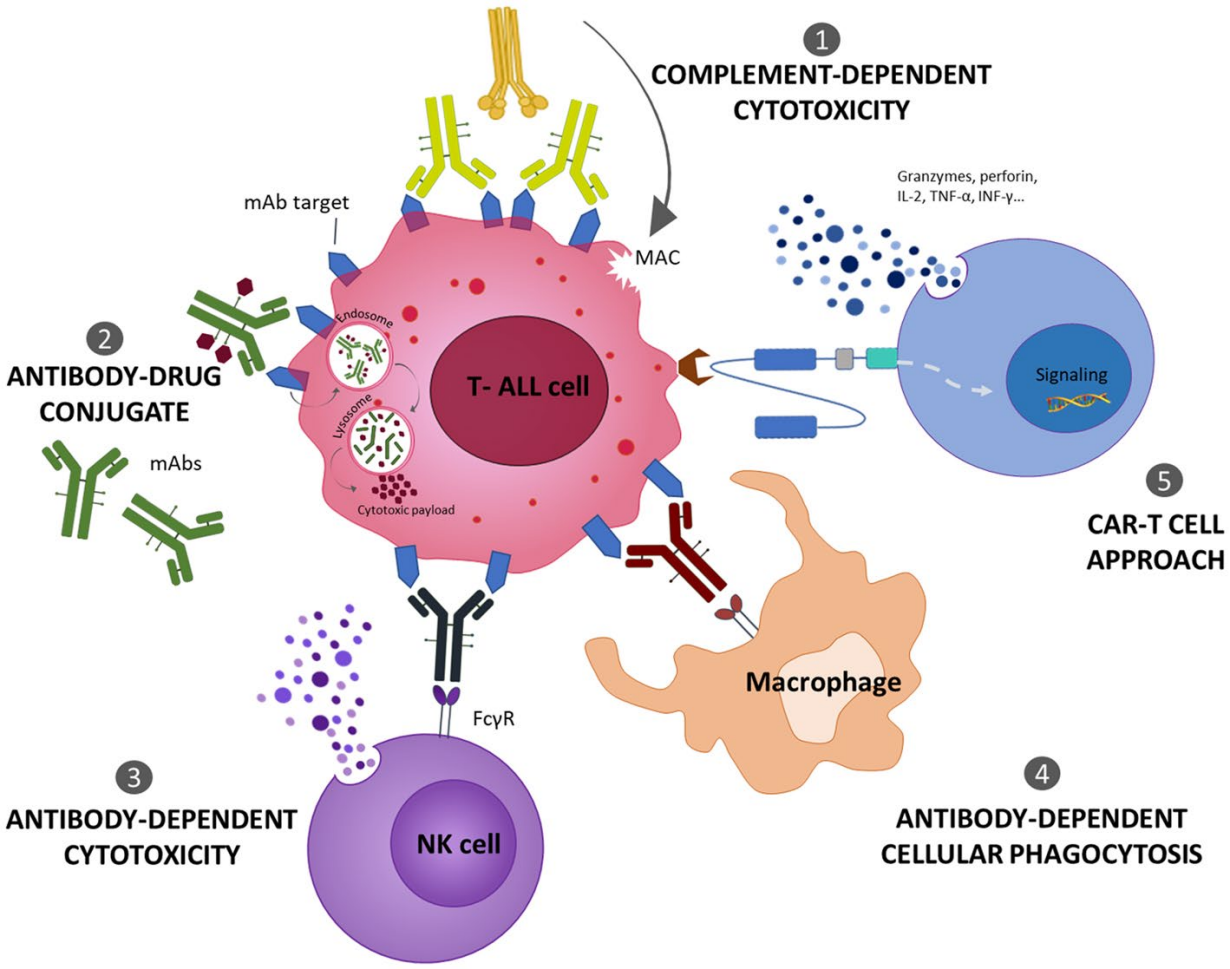
Mechanisms of action of antibodies targeting T-ALL cell and CAR-T cell approach. Antibodies induce tumor cell-killing through different mechanisms: (1) initiating the complement cascade [complement-dependent cytotoxicity (CDC)]; (2) delivering cytotoxic drugs to be internalised in tumor cells [antibody–drug conjugation (ADC)]; (3) activating immune effector cells, namely NK cells [antibody-dependent cytotoxicity (ADCC)] or (4) macrophages [antibody-dependent cellular phagocytosis (ADCP)]. On the other hand, CAR-T cells act (5) recognizing and killing cancer cells through the release of inflammatory cytokines and cytolytic molecules

mAbs bind cell surface antigens and can either prevent the interaction with ligands or inhibit receptor clustering and stimulation, leading to apoptosis of target cells [31]. Moreover, by binding of the Fc regions to Fc gamma receptors (FcγRs) on the surface of immune cells or complement factors, mAb therapeutics activate effector mechanisms (Fig. 1), as antibody-dependent cellular cytotoxicity (ADCC), antibody-dependent cellular phagocytosis (ADCP), and complement-dependent cytotoxicity (CDC), inducing the killing of antigen-expressing cells [32–34]. Another mAb-based immunotherapy relies on antibody–drug conjugates (ADCs) that are internalized and release cytotoxic agents into targeted cells [35–38]. The recent availability of humanized mAbs and the conjugation to powerful drugs have led to the approval by the FDA of ADC targeting CD33 in acute myeloid leukemia (AML) (gemtuzumab ozogamicin, Mylotarg®), CD30 in T-cell lymphoma (TCL) and Hodgkin lymphoma (brentuximab vedotin, Adcetris®), and CD22 in B-ALL patients (inotuzumab ozogamicin, Besponsa®) [39, 40]. Since the approval of Mylotarg® in 2020, a total of 14 ADCs reached the market worldwide [41]. Presently, several ADC are under investigation in clinical trials for T-ALL treatment [39].

More recently, immunotherapy with CAR-T cells has emerged as a powerful strategy for relapsed/refractory haematopoietic malignancies such as B-ALL [42, 43]. Peripheral blood T-cells are ex vivo engineered to express a receptor specific for a surface antigen/epitope expressed by cancer cells. These engineered immune effectors are finally amplified to be reinfused in the patient. CAR-T cells can recognize and kill tumor cells in a major histocompatibility complex (MHC)-independent manner [44].

## BTCEs: a new hope for T-ALL immunotherapy

**Fig. 2:**
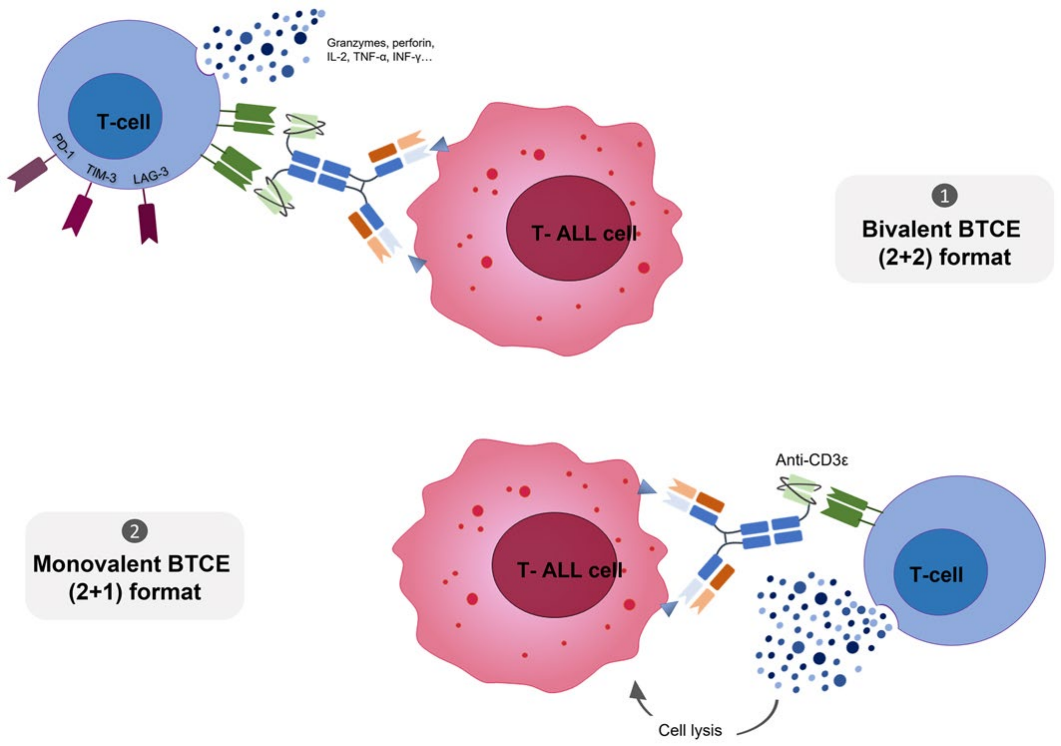
Mechanisms of action of BTCEs. (1) Bivalent BTCEs (bBTCEs), characterized by two arms binding CD3Ɛ (2 + 2 format), re-direct T lymphocytes against T-ALL cells expressing the target antigen. (2) Monovalent BTCEs (mBTCEs), constituted by one arm binding CD3Ɛ (2 + 1 format), empower cytotoxic effect on T-ALL cells compared to bBTCEs and limit T-cell exhaustion

BTCEs have recently emerged as effective and off-the-shelf therapeutics to induce an immunologic synapse between a tumor-associated antigen (TAAs) expressed by cancer cells and cytotoxic immune effectors. For this aim, a BTCE plays a cell-bridging function completely absent in parent mAbs. There are different species of BTCEs, ranging from small-scale proteins, characterized by two single chain variable fragments (scFv), to longer asymmetric or symmetric immunoglobulin G (IgG)-like molecules [45]. IgG-like constructs offer different pharmacodynamic and pharmacokinetic advantages: a) the presence of neonatal Fc receptor (FcRn) protects IgG-like BTCE from rapid degradation and confers long plasma half-life (days) as compared to the shorter plasma half-life (hours) of Fc-fragment lacking BTCE that, instead, need continuous infusion; b) the presence of bivalent binding domains for TAAs increases the avidity and the selective recognition of antigen-expressing T-ALL cells [45–47]. Furthermore, regarding the activation moiety of effector T cells, at least two different IgG-like BTCEs format could be distinguished: bivalent BTCE (2+2), with two arms binding to a TAA and two arms binding to CD3ε, and monovalent BTCE (2+1), with two arms binding to a TAA and one arm binding to CD3ε (Fig. 2). Although both constructs were effective against cancer preclinical models, higher anti-tumor activity has been observed with monovalent BTCEs mainly due to the mitigation of nonspecific T-cell activation by CD3 cross-linking.

The discovery of T-ALL selective antigens might provide effective therapeutic options for the treatment of this orphan disease by a BTCE-based approach.

## Challenges in immunotherapy for T-ALL

The above-described strategies have been mostly based on the targeting of the main T lineage targets, such as CD1a, CD5, CD7, CD38, that, indeed, are shared by the normal T cell compartment and produce T-cell fratricide effects and severe T-cell aplasia as consequence of on-target/off-tumor cytotoxicity. These events, together with novel toxicities associated to T-cell activation, such as the Cytokine Release Syndrome (CRS) and the Immune effector Cell-Associated Neurotoxicity Syndrome (ICANS), which is more common in BTCE-treated patients, represent therefore the main issues to be overcome in the next future for a clinically meaningful application of immunotherapy in T-ALL.

### Strategies to prevent fratricide effects and T-cell aplasia

While shared expression of B cell lineage antigens between normal and malignant cells results in B cell aplasia, which can be easily managed with immunoglobulin administration, T cell aplasia can result in severe and life-threatening immunosuppression further exacerbated by the absence of effective T cell replacement options.

Currently, to overcome these roadblocks, several strategies are under investigation. To avoid T cell aplasia, CAR-T could be generated by transducing T cells with adeno-associated viral vectors, which confers transient CAR-T expression due to lack of genomic integration [48]. Similarly, non-viral CAR-T delivery through mRNA electroporation confers only transient CAR-T function which can mitigate T cell aplasia with prompt rescue. However, the heterogeneous expression and the low persistence of CAR-T may impair their antitumor activity [49].

The incorporation of suicide genes and safety switches could prevent T cell aplasia, by selective cell-death induction of transduced T cell while sparing normal T lymphocytes. Examples of these approaches are represented by metabolic activation of non-toxic compounds such the herpes simplex virus thymidine kinase (HSV-TK) coupled with the use of ganciclovir [50], use of inducible cas9 (iCas9) [51] or the expression of antigen genes in CAR vectors combined with the use of mAbs resulting in ADCC of transduced cells [52].

Beyond T-cell aplasia, the shared expression of antigens between normal and neoplastic T-cells makes that CAR T cells will inevitably kill themselves, an effect called fratricide, with consequent anti-tumor immunity impairment. To overcome this relevant issue, different approaches have been developed. One of these is based on Tet-OFF inducible expression system, which can be used to circumvent the T cell fratricide activity by temporally controlling CAR-T expression. In this model, the expression of a CAR is repressed in the presence of doxycycline in vitro while is restored with a doxycycline-free environment allowing CAR-mediated cytotoxicity [53].

Another way to prevent the fratricide is the genome editing of the tumor antigen in CAR-T cells using DNA nuclease technology such as CRISPR-Cas9. This approach was successfully used to edit CD7 expression in CAR-T lymphocytes, enabling the expansion of CAR T cells without affecting antitumor function [54].

Protein Expression Blockers (PEBL) technology was also used to limit T cell fratricide by preventing the surface expression of CD7. In detail, CD7 down-regulation on CAR-T was achieved using a scFv directed against CD7 coupled with an ER (endoplasmic reticulum) / Golgi-retention motif which ensures CD7 anchoring in the ER and/or Golgi [55].

Finally, engineering other cytotoxic immune cells such as primary NK (natural killer) or NK-92 cells lines could be another suitable option to prevent T cell fratricide, considering that these cells do not express T cell associated antigen endogenously [56].

## T-cell surface antigens as immunotherapeutic target for T-ALL

### CD1a

CD1a is a surface glycoprotein promoting antigen presentation to specialized T cells and it is expressed on approximately 40% of cortical-derived T-ALL patients [57]. On normal tissues, CD1a is present on transient cortical thymocytes, skin Langerhans cells and a subset of circulating myeloid dendritic cells, but it is not expressed by mature T cells [10, 58–61]. This pattern of expression makes the targeting of CD1a less prone to fratricide effects and potentially reduces the risk of on target/off-tumor side effects [48]. To date, a few studies have contributed to define the in vivo role of CD1a, principally because this CD1 isoform is lacking in mice [62]. However, a recent study performed on CD1a transgenic mice showed that the expression of this molecule is strictly related to the pathogenesis of poison-induced cutaneous diseases [60]. Authors reported the absence of adverse effects following the treatment of induced skin inflammation using anti-CD1a antibodies, thus supporting the idea that CD1a is a safe and attractive target for cortical T-ALL (coT-ALL) subtypes. In this context, an anti-CD1a mAb named CR2113, has been demonstrated to induce potent ADCC in CD1a-expressing cell lines and T-ALL primary samples in vitro, which translated in anti-tumor activity against CD1a-expressing xenografts [63].

Recently, a second-generation (4-1BB costimulatory domain) CD1a CAR-T cells showing no fratricide effects and long-term persistence, was developed [61]. Since CD1a is not expressed by peripheral blood T-cells, the target gene on effector cells did not need to be knocked out. Through in vitro studies, the authors demonstrated the specific cytotoxicity of CD1a CAR-T cells against CD1a+ T-ALL cell lines and primary blasts, while studies on a patient-derived xenograft model of cortical coT-ALL confirmed their potent anti-leukemic activity. Although not applicable to all T-cell malignancies, CAR-T cells for CD1a targeting may represent a successful strategy in the specific subset of coT-ALL patients. A phase I clinical trial is presently ongoing to study its efficacy and safety [48].

More recently, starting from the development of a novel anti-CD1a mAb, named UMG2, a monovalent BTCE that simultaneously binds CD1a on T-ALL cells and CD3ε on T-lymphocytes (CD1a x CD3ε) has been generated [64]. The authors demonstrated that UMG2 binds a previously uncharacterized CD1a epitope, with a strong reactivity on cortical T-ALL cells, while no binding was found on normal peripheral blood cells. CD1a x CD3ε BTCE produced significant in vitro T-cell mediated cytotoxicity against CD1a expressing T-ALL cells and inhibited the growth of human T-ALL xenografts in vivo. These data suggest that CD1a x CD3ε BTCE may be suitable for clinical development as an innovative immunotherapeutic tool for the treatment of CD1a-expressing cortical-derived T-ALL patients.

### CD3

CD3 is a multimeric protein complex highly expressed in mature T-cell lymphomas and mature T-ALL, and its cytoplasmic expression is considered a diagnostic marker for immature T-ALLs. It is expressed by haematopoietic cells and is involved in the association with the T-cell receptor (TCR) on T-cells and thymocytes surface [65].

The first anti-CD3 mAb available for therapy in humans was Muromonab, approved in 1985 for the treatment of organ transplant rejection [66]. The antitumor activity of anti-CD3 mAbs in T-ALL has been subsequently reported. Indeed, the activation of TCR signalling by the treatment with anti-CD3 mAbs induced leukemic cell death in T-ALL mouse models [67]. A further study demonstrated the preclinical efficacy of humanized non-FcγR-binding anti-CD3 mAbs in xenograft models of T-ALL. The anti-leukemic effects and host survival was increased by the combination of these antibodies with chemotherapy [68].

CD3 was also investigated as a potential target in TCL using immunotoxin-loaded anti-CD3 mAbs: the treatment was well tolerated and induced the partial remission of 2 of 5 patients [69]. The promising results led to the development of anti-CD3 CAR-NK cells. The expression of a third-generation anti-CD3 CAR, incorporating CD28 and 4-1BB costimulatory domains, in the NK-92 cell line induced the in vitro and in vivo killing of CD3+ lymphoma cells, primary T cells and T-ALL cells [70]. A second-generation anti-CD3 CAR, containing a 4-1BB costimulatory domain, demonstrated in vitro antitumor activity against CD3+ primary T cells and childhood T-ALL cells [71]. Finally, a novel strategy, based on the use of a second-generation (4-1BB costimulatory domain) CD3 CAR, was developed to generate allogenic T cells not expressing TCRαβ [72].

Although the encouraging results, the development of a CD3-based immunotherapy against T-ALL is strongly hampered by the risk of severe immune-depression and fratricide effects, due to CD3 wide expression on normal T cells.

### CD4

CD4 was one of the first targets investigated for therapeutic purposes since it is expressed by a wide range of mature T-cell lymphomas and a subset of T-ALLs [73]. In normal cells, it is expressed by about 80% of thymocytes and in more than 50% of peripheral blood T-lymphocytes [74, 75], and is involved in T-cell activation, acting as a co-receptor for the TCR [76].

Since the use of anti-CD4 mAbs showed a reversible depletion of CD4+ cells in T-cell lymphoma patients without inducing immunosuppression [77–79], third-generation anti-CD4 CARs (containing 4-1BB and CD28 costimulatory domains) were then developed, demonstrating in vitro and in vivo preclinical efficacy [73, 80]. A phase I clinical trial (NCT04162340) is evaluating the safety and the antitumor efficacy of anti-CD4 CAR-T cells in T-cell malignancies, including T-ALL. Starting from the same CD4 CAR structure and inserting a natural safety switch based on alemtuzumab (anti-CD52 mAb), Ma et al. extended these studies [81]. Indeed, alemtuzumab, recognizing CD52 on CAR-T cells, was able to remove CD4 CAR-T cells after tumor depletion, thus limiting toxicity. However, further studies are needed to find the best dosage of alemtuzumab that leads to the removal of most CAR-T cells without compromising their antitumor efficacy.

### CD5

CD5 is a type-I transmembrane glycoprotein of 67 kDa, belonging to scavenger receptor cysteine-rich superfamily [82]. Since it is expressed in about 80% of T-ALLs and T-cell lymphomas, CD5 represents a surface marker of malignant T-cells [83]. In normal cells, its expression is found on thymocytes, peripheral T cells and a subset of B-cells [48, 84]; but it has been reported to be expressed on other immune-cell subtypes such as macrophages and dendritic cells [85, 86]. CD5 participates in T-cell development and function, acting as a negative regulator of TCR signalling [82, 87, 88]. It protects against autoimmunity, preventing T-cells from uncontrolled self-reactivity [88].

Some clinical trials reported tumor cells depletion in patients with T-ALL and cutaneous T-cell lymphoma after treatment with toxin-conjugated anti-CD5 mAbs [89–91]. Importantly, no severe side effects were observed during these studies. Thus, the development of CAR-T cells directed against this surface marker seemed a safe and useful strategy for the treatment of T cell malignancies.

As reported in a preclinical work, T cells engineered to express a second-generation CD5 CAR, incorporating the CD28 costimulatory domain, showed low surface CD5 level [92]. This produced poor fratricide and allowed ex vivo expansion. T-ALL and T-cell lymphoma cells were successfully eliminated in vitro, and disease progression was controlled in vivo in two different CD5^+^ T-ALL models. Considering the promising results of this study, an ongoing clinical trial (NCT03081910) is evaluating the efficacy and safety of these CARs.

Other studies demonstrated that also the use of CD5-negative cells and CD5-CRISPR-Cas9-edited T-cells could overcome the fratricide related to the innate expression of CD5 promoting the antitumor efficacy against T-cell leukemia cell lines [93, 94]. A research group investigated CD5-CAR-edited NK cells, using an approach based on CAR incorporating a costimulatory domain 2B4 (CD25-2B4-CAR NK-92 cells), demonstrating a great efficacy against CD5+ malignant cells either in vitro and in vivo [95].

Recently, a bioepitopic CAR with fully human heavy-chain variable (FHV_H_) domains for the recognition of different epitopes of CD5, was developed [96]. Also in this case, fratricide in CD5 CAR-T cells was prevented through CD5 knockout via CRISPR-Cas9 genome editing. An enhanced and prolonged efficacy was confirmed through in vitro and in vivo studies.

In another study, T cells were transduced to express third generation CD5 CARs including the CD28 and 4-1BB costimulatory domains [97]. The CD5 CAR-T cells efficiently lysed CD5+ malignant T cell lines and primary cells in vitro, and tumor progression was controlled in vivo. Unfortunately, this construct also induced toxicity against normal T cells.

### CD7

CD7 is a transmembrane glycoprotein of 40 kDa widely expressed during T-cell differentiation from progenitors or on mature T and NK cells. It is expressed at high levels in lymphoblastic T-cell leukemia, lymphomas and in a subset of peripheral T-cell lymphomas (PTCL) [98, 99]. It plays a significant role in T-cell activation and interactions with other immune cells, but it does not seem to have a pivotal role in T-cell development or function. Indeed, studies performed on murine models showed that T cells lacking in CD7 reported unaltered homeostasis and development, also retaining their short-term effector function and antitumor activity [54, 100]. The activity of an anti-CD7 mAb-ricin A chain immunotoxin in patients with T-cell lymphomas and leukemia, was evaluated [101]. Although no heavy CD7-related toxicity was observed, the antitumor effects were moderate, due to the low activity of murine antibodies on human patients [99].

Further studies have been performed on CD7 as a promising target for CAR-T cell therapies. However, unlike CD5, the incomplete down-regulation of CD7 on engineered CAR-T cells causes fratricide and prevents their ex vivo expansion [102]. Then, to avoid self-antigen targeting in CAR-T cells, CD7 surface expression must be abrogated. To avoid fratricide effect, another intriguing strategy is based on naturally occurred CD7-negative T cells as a source for CAR-T generation. The CD7-negative population can be easily isolated from bulk T cells using a two-step magnetic isolation protocol. This T-cell subpopulation, after the CAR-T engineering, is characterized by a favourable biological profile. Indeed, they are predominantly CD4+ effector memory and CD4+ and terminally differentiated effector memory cells with a preserved expansion activity and viability. Moreover CD7 CAR-T cells are characterized by an effective antitumor immune response, as demonstrated by their ability to kill both CD7 and CD19 expressing haematological malignant cells [103].

Over the past few years, three other groups reported the efficacy of CD7-specific CARs in preclinical models of T-cell malignancies. All these studies considered the abrogation of surface expression of CD7, simply by modifying the CD7 gene or inhibiting the CD7 protein trafficking to the surface of cells [54, 55, 104]. In detail, PEBLs technology prevents CD7 surface expression retaining newly formed CD7 in the ER or Golgi [104–106]. TALEN and CRISPR systems are other well-known strategies for the knocking-out of the target gene [102]. This approach has been considered in an ongoing Phase I clinical trial (NCT03690011) which was based on the investigation of the effects related to the presence of CD7-CRISPR-Cas9-edited CD7 CAR-T cells in patients with CD7+ T-cell malignancies, including T-ALL [99]. In another study, anti-CD7 CAR-T cells using CRISPR/Cas9 and lentiviral transduction approaches (TRAC-/-CD7-/-, CD7 UCAR) against T-ALL [107] were developed. The CD7 UCAR-T cells could induce the killing of primary T-ALL cells in vitro, with high degranulation level and proinflammatory cytokines release, and were able to reduce tumor growth and increase mice survival in vivo.

The potential use of a third generation (CD28 and 4-1BB co-stimulatory domains) CD7-specific uCAR for the treatment of r/r T-ALL and non-Hodgkin’s T-cell lymphoma, was described [104]. The abrogation of both CD7 and T-cell receptor alpha chain (TRAC) was induced using CRISPR/Cas9 technology. Since abrogation leads to impaired TCR signalling, this strategy could prevent either fratricide or T-cell-mediated graft-versus-host diseases. CD7 uCAR-T cells proved to be effective against human T-ALL cell lines and primary T-ALL cells. However, a short lifespan is expected from these allogenic CAR-T cells, related to the immune reconstitution of the host. This effect prevents cells aplasia, but it could be not effective in cancer recurrence [102]. Moreover, both strategies based on CRISPR-Cas9 gene editing technology or involving ER retention of CD7 molecules require genetic T-cell engineering, which can lead to long-term side effects [108]. For this reason, current trends involve the culturing of anti-CD7 CAR-T cells with a recombinant anti-CD7 antibody as a safe and cost-effective strategy in T-ALL therapy [109]. Recently, the addition of a blocking antibody has been demonstrated to increase T-cell viability and expansion and prevent cell exhaustion, thus obtaining long-lasting and effective anti-CD7 CAR-T cells for the treatment of T-cell malignancies. In a phase I clinical trial, anti-CD7 CAR-T cells, edited from stem cell transplantation, were administered in 20 patients with r/r T-ALL demonstrating great efficacy and low toxicity [110]. Then, a multicentre phase II trial of CD7 CAR-T cells derived from donors is now ongoing (NCT04689659).

In the last few years, researchers have focused on different targeting domains for CARs, such as nanobodies, peptides and other ligands [111–115]. Thanks to their small size, easy production, high stability and targeting selectivity, nanobodies emerged as promising strategies [112]. In this regard, several pre-clinical and clinical studies have reported a comparable efficacy between scFV-based CAR-Ts and nanobody-based CAR-Ts [116]. Tandem CD7 nanobody combined with an endoplasmic reticulum/Golgi-retention motif peptide able to prevent fratricide thanks to its CD7 blocking effect, was reported [117]. Autologous nanobody-derived fratricide-resistant CD7 CAR-T cells proved a long-lasting antitumor activity in r/r T-ALL with manageable toxic effect. The manufacturing of nanobody derived CD7 CAR-T cells also induced haematological and extramedullary remission in a 11-year-old male with TP53 mutated r/r ETP-ALL/LBL [118]. The patient was enrolled in an anti-CD7 CAR-T clinical trial (NCT04785833) after failure of 4 lines of salvage therapies. Grade 3 CRS and macrophage activation syndrome were observed, but these side effects were reversible.

These approaches often require genetic editing to ablate the CD7 gene or block CD7 cell expression and induce the selective targeting of CD7 on the CAR-T cells. A novel approach focuses on the masking of natural CD7 molecules, by CD7-targeting CAR. The relevant reduction in CD7 accessible epitopes allowed to overcome the fratricide challenge. In a recent study, this approach showed an improved efficacy, compared to that recorded from sorted CD7-negative and CD7 knocked-out CAR-T cells, thanks to the greater amount of CAR^+^ cells obtained and higher percentages of CD8^+^ central memory T cells [119]. Based on these results, a first-in-human phase 1 trial (NCT04572308) was performed involving 20 patients, 14 of them with r/r T-ALL. Patients were treated with naturally selected CD7 CAR-T cells, and most of them achieved complete remission reporting no relevant side effects. However, a larger number of patients and longer follow-up are required to validate these results.

### CD25

CD25, also known as IL2R, represents the alpha chain portion of interleukin 2 receptor. It is expressed on activated T-cells and participates in their proliferation and death, as well as in maintenance of regulatory T-cells. Previous studies reported that this receptor plays a key role in refractory ALL or AML [120, 121]. In a single case-report basiliximab, an anti-CD25 mAb, showed its efficacy on a 5-year-old patient with T-ALL. In detail, the patient reported a clinical status consistent with the febrile ulceronecrotic Mucha-Habermann disease, which prevented the intensification of chemotherapy due to the relative superinfection. In this context, the co-treatment with basiliximab improved cutaneous eruptions, allowing to intensify chemotherapy and proceed with the bone marrow transplantation [122]. This event gave hope for the use of mAbs targeting CD25 as adjuvants for T-ALL therapy. However, to date there are no clinical trials ongoing using this molecule in the treatment of T-ALL patients.

### CD30

CD30 is a member of the tumor necrosis factor receptor (TNFR) superfamily, expressed not only in Hodgkin's lymphoma but also in other haematological diseases such as anaplastic large cell lymphomas, cutaneous and peripheral T-cell lymphomas, adult T-cell leukemia/lymphoma and diffuse large B-cell lymphomas. In normal cells, it is present on T helper cells, on a subset of CD8+ T cells and on a subset of B lymphocytes [123].

Since in preclinical studies anti-CD30 treatment has been found to be effective in removing cancer cells without affecting normal lymphopoiesis, CD30 could be considered a potential candidate for immunotherapeutic targeting [124, 125]. In this context a CD30 antibody-drug conjugate, brentuximab vedotin (BV), has achieved therapeutic approval for peripheral T cell lymphoma (PTCL) and cutaneous T cell lymphoma (CTCL) and Hodgkin lymphoma, both as monotherapy or in combination with standard chemotherapy [126, 127]. Furthermore, CD30 CAR-T cells have been evaluated in clinical trials for the treatment of r/r Hodgkin lymphoma and anaplastic large cell lymphoma patients, showing good tolerability but modest efficacy [128, 129].

Since CD30 is expressed on 38% of cases of T-ALL and is up-regulated during high-dose chemotherapy [130], anti-CD30-based immunotherapy could be a promising strategy for T-ALL patients. However, to our knowledge no clinical studies are ongoing for the evaluation of CD30 targeting in T-ALL patients.

### CD38

CD38 is a type II transmembrane glycoprotein of 45 kDa playing a dual role as a receptor and cell surface enzyme (ectoenzyme) [131]. This receptor is expressed at early stages of B and T-cell development, it is downregulated in mature naïve lymphocytes, re-expressed after T-cell activation, and finally lost in the T-cell memory compartment [10, 132]. As an ectoenzyme it enzymatically converts nicotinamide adenine dinucleotide (NAD) to cyclic ADP-ribose, an important calcium-mobilizing second messenger [133]. In T cells, CD38 signalling is associated with TCR function, inducing the activation of intracellular molecules as Zap70, Erk and Akt/PKB [134]. Its ligation induces death of T-cell precursors and contributes to the selection of thymocytes in the thymus. CD38 is also implicated in the regulation of lymphocyte adhesion to endothelial cells and regulates in vitro cytotoxic T-cell activity.

Since it is widely expressed in haematological tumors, CD38 targeting may provide a rationale for novel therapeutic strategies involving CAR-T cells [135, 136].

Recently, CD38 expression has been demonstrated by flow cytometry in different T-ALL subtypes [137]. On these findings, T-ALL patients who relapse or do not respond to conventional therapies could be treated with antiCD38-mAbs. Moreover, the treatment with all-trans retinoic acid (ATRA), clinically used for acute promyelocytic leukemia (APL), can induce CD38 upregulation in CD38^low^ adult T-cell leukemia (ATL) cells, making them susceptible to CD38 CAR-T cells [138]. The combining effect of ATRA and INF-α eradicated more than 95% of these leukemic cells. However, the safety of this treatment still needs to be clarified.

Interestingly, preclinical efficacy of anti-CD38 daratumumab in T-ALL has been reported [139]. CD38 was identified in ETP-ALL and in non-ETP-ALL patient leukemic cells. It was found that almost all T-ALL patient-derived xenografts (PDXs) were sensitive to daratumumab. Furthermore, it was well tolerated by a 19-yr old patient with refractory ETP-ALL and induced a temporary reduction of T-cell lymphoblasts in the bone marrow [140]. Finally, daratumumab eradicated MRD in some T-ALL patients suffering relapse after allogeneic stem cell transplantation [141]. Recently, in vitro and in vivo effects of ATRA in combination with CD38-targeting CAR-T cells or daratumumab, were evaluated [142]. At first, authors proved the robust effect of their CD38 CAR-T cells on lymphoid cells expressing high levels of CD38, such as T-ALL. Interestingly, they found that ATRA significantly increased the expression of CD38 in cancer cell lines with low expression of this marker, thus producing a synergistic effect with CAR-T cells or daratumumab and expanding the possibility of considering CD38 as an efficient target in a wider type of lymphoid malignancies as well. By another approach, combining daratumumab with CD47 inhibitor has been demonstrated to increase phagocytosis of T-ALL cells [143].

The preclinical activity of ISB 1442, a human anti-CD38 and anti-CD47 bispecific antibody currently in a Phase 1/2 clinical trial for r/r MM (NCT05427812), was evaluated in AML and T-ALL [144]. The co-targeting of CD38 and CD47 by a single antibody with increased Fc effector functions led to a strong in vitro phagocytosis, especially in CD38 over-expressing cell lines, and to a potent ADCC.

Two ongoing clinical trials are evaluating the effectiveness of daratumumab (NCT03384654) and isatuximab (NCT03860844) for r/r B or T-ALL. Anti CD38 immunotherapies using ATRA have been evaluated in a recent clinical trial (NCTC02751255) in combination with daratumumab in MM. However, a limited activity emerged in patients due to a temporary increase of CD38 antigen expression (145).

Recently, a novel CD38 x CD3 BTCE with Fc domain engineered to limit Fcγ receptor binding and non-specific T-cell activation, is currently being investigated in a Phase 1 study for patients with r/r T-ALL and AML (NCT05038644) [146].

### CD43

CD43 is a mucin-like type I transmembrane protein expressed on haematopoietic cells, such as T-lymphocytes, granulocytes, NK cells, monocytes, platelets, and haematopoietic stem cells, but not on mature erythrocytes and B-cell subpopulations. It is involved in a variety of functions including cell activation, proliferation, adhesion, and invasion and may be implicated in immune response by modulating cell growth, survival, and apoptosis [147]. Several studies reported the association between CD43 and cancer: CD43 glycoforms were detected in different tumors also of non-haematopoietic origin but not in the normal tissues [148].

The targeting of a unique epitope of CD43, named UMG1, has been recently proposed as a selective strategy for T-ALL treatment [149]. This epitope was found expressed in approximately 50% of cases of T-ALL patient-derived samples (82% of EGIL TIII patients). Importantly, no antigen expression was found in normal tissues, except for cortical thymocytes and a very small subpopulation of peripheral blood T lymphocytes (<5%), thus excluding the fratricide risk and, also, the targeting of other components of the normal hematopoietic and non-hematopoietic compartments. The expression profile of this specific epitope appears, therefore, highly promising, and significantly differs from the canonical CD43 pattern of expression, as characterized by expression of other epitopes already described, whose targeting, instead, would result in relevant on target/off tumor side effects.

An afucosylated humanized mAb was produced with a long selection process from a previous murine mAb [150–152] and, on this basis, two different IgG-like BTCEs against UMG1 have been developed: bivalent UMG1-BTCE (2+2), with two arms binding to CD3ε, and monovalent UMG1-BTCE (2+1), with one arm binding to CD3ε. Importantly, these approaches resulted in significant anti-leukemic in vitro and in vivo activity, which was higher for BTCEs treatment as compared to afucosylated mAb. In particular, both BTCE constructs were effective against T-ALL preclinical models in a clinical range of concentrations similar to Blinatumomab, the first BTCE approved for B neoplasms [153]. However, higher anti-leukemic activity was observed with monovalent BTCE, mainly due to reduced T-lymphocytes exhaustion [149].

These results suggest that the targeting of this epitope may represent a safe and effective new therapeutic option against T-ALL to be explored in the front-line as well as maintenance treatment, in a first in human clinical trial.

### CD44

CD44 is a type I single-span transmembrane glycoprotein that acts as a cell surface receptor for a component of the extracellular matrix, the hyaluronan. The CD44 gene has various isoforms, differentially expressed in human tissues [154]. The standard isoform (CD44s) is the most abundant in the haematopoietic compartment, whereas variant isoforms (CD44v) are present in some populations of epithelial and haematopoietic cells, particularly during development, in some types of carcinoma and after lymphocyte activation [155]. In cancer cells of epithelial origin, the CD44v6 isoform is usually overexpressed, playing a key role in migration, metastasis and chemoresistance. CD44 was thought to be involved in the homing of lymphocytes into lymph nodes [156] and in the anchoring of haematopoietic precursors [157] and leukemic cells [158] within the bone marrow niche. Moreover, it is highly expressed in the earliest human CD34+ T-cell precursors of thymus and downregulated on T-cell commitment and during T-cell development, while it is preserved in myeloid-primed intra-thymic progenitors with dendritic cell potential [159].

CD44 has been well studied in solid tumors for its function in maintaining cancer-initiating cell properties [160], but its contribution in haematological malignancies has been formally demonstrated very recently [158]. CD44 is a direct transcriptional target of NOTCH1, which was upregulated in NOTCH1-induced preleukemic blasts [161]. Though CD44 expression is aberrant in leukemic blasts of T-ALL patients, no correlation with prognosis and survival has been demonstrated [162]. Since it is also overexpressed in NOTCH1-induced T-ALL leukemic cells treated with chemotherapeutic drugs [163], CD44 may be considered as a valuable target for new therapies against relapsed or chemo resistant T-ALLs. However, to date, no clinical trial has been activated for anti-CD44 therapy in T-ALL patients.

### CD52

CD52 is a surface glycoprotein, that is not present in normal haematopoietic progenitors but is widely expressed in B- and T-cells, lymphocytes, monocytes, and macrophages [164]. High expression levels of CD52 are also reported in some cases of T-ALL; however, pre-T leukemic blasts showed lower expression than mature cells, indicating that immunotherapeutic approaches involving CD52 might be limited to mature subtypes [164–166].

CD52 function is not yet completely understood but some studies reported that it can induce T-cell activation and stimulate the production of CD4+ regulatory T-cells, thus activating immunosuppressive mechanisms [167, 168]. Many trials investigated the efficacy of alemtuzumab (CAMPATH-1H), as a mAb targeting CD52, on T-cell diseases including T-ALL, with poor results [169, 170]. The latest trial, involving patients with more than 10% of CD52+ lymphoblasts, focused on the efficacy and safety of the combination of alemtuzumab with chemotherapy for the eradication of MRD in T-ALL patients. However, these studies demonstrated no advantage over the other available therapies and the occurrence of many side effects that led to the termination of trials involving CD52 [171]. Other approaches involved CD52-knockout CAR-T cells, which were obtained through TALEN technology. A depletion of patient T-cells using alemtuzumab and a subsequent infusion of the engineered T-cells have been proposed in the attempt to enhance their engraftment and retention, thus representing a promising treatment for T-ALL [172, 173].

### CD99

CD99 is an O-glycosylated transmembrane protein present on leukocytes and involved in cell adhesion, T-cell rosetting and trans-endothelial migration [174]. Preclinical studies reported that anti-CD99 mAbs induced caspase-independent cell death of AML cell lines and primary blasts [175], T-cell lines [176], and TEL/AML1-positive ALL and normal B-cell precursors [177].

CD99 expression is higher in T-ALL cells than in haematopoietic stem cells and normal T-cells [178] and its detection by flow cytometry is useful to detect MRD in T-ALL patients [179], representing a promising target. An in vitro study showed that the treatment of T- and B-cell lines with anti-CD99 antibodies upregulated heat shock protein 70 (HSP70), increasing NK-dependent cytotoxicity [180]. Moreover, Shi et al. demonstrated that CAR-T cells targeting CD99 specifically recognized and eradicated T-ALL cell lines and primary tumor cells without normal blood cells toxicity [181]. Thus, CD99 might represent a candidate for alternative therapeutic approaches against T-ALL to be validated on early clinical setting.

### CD194

CD194 (CCR4) is the receptor for two chemokines, CCL17 and CCL22, expressed on Type 2 helper T-cells (Th2) and Treg. Its expression is high in mature T-cells, and it has been shown to be implicated in the homing of leukocytes to inflamed sites [182]. Mogamulizumab is an anti-CCR4 humanized antibody, which has been approved in Japan since 2014 for patients with adult T-cell leukemia. This drug showed a great potential in T-ALL therapy as an adjuvant for chemotherapy and it has been considered as a promising strategy for transplant-ineligible patients with adult T-cell leukemia or lymphoma [183]. Furthermore, an interesting approach based on CCR4 CAR-T cells displayed antigen-dependent potent cytotoxicity against patient-derived cell lines of T-cell lymphoma, thus suggesting the feasibility of such approach also for CCR4-expressing T-ALL patients [184].

### CCR9

Another promising target for T-ALL immunotherapy is represented by a chemokine receptor CCR9. Indeed, while CCR9 is expressed only on a small fraction of normal T cells (<5%), it could be found in >70% of cases of T-ALL, including >85% of relapsed/refractory disease. More recently, CAR-T-cells targeting CCR9 have been demonstrated to induce high anti-leukemic in vitro and in vivo activity, without fratricide effects. Thus, such an approach holds the promise to be a highly effective treatment strategy for T-ALL, avoiding detrimental effects of T cell aplasia or an expensive approach of genome engineering for the prevention of fratricide activity [185]***.***

### CXCR4

CXCR4 is a chemokine receptor belonging to the superfamily of G protein-coupled receptors and it is implicated in several regulating processes, including hematopoiesis and immune response [186]. CXCR4 interacts with CXCL12 (CXC motif chemokine 12)/Stromal cell-derived factor-1 (SDF-1), that is a chemokine with a critical role for cells escaping from thymus to bone marrow, leukemia initiating cells and trans-endothelial migration [187, 188]. For this reason, studies blocking CXCR4 by mAbs [189] or small molecule [190] have shown a great potential effect on leukemia maintenance and progression [191]. A key role of CXCR4 in T-ALL pathogenesis was demonstrated by the inhibition of the receptor expression on NOTCH1-induced T-ALL mice, using a short hairpin RNA (shRNA) [192]. Authors reported an impaired tumor cells migration and an increased cell death in vitro and efficacy in vivo.

A phase II trial assessing the efficacy and safety of a small synthetic peptide targeting CXCR4, BL8040, in combination with Nelarabine for the treatment of r/r T-ALL patients (NCT02763384), is ongoing.

### IL7R

Interleukin-7 (IL-7) receptor is characterized by a heterodimeric structure with a specific α chain (IL7Rα) and a gamma portion, which is common to numerous cytokine receptors [193]. IL-7 was initially defined as a growth factor for B-cells, but its involvement in cell growth was demonstrated to be also important for T-cells. Through the induction of IL7R signalling, IL-7 defines the maturation of T-cells from the thymus [194, 195]. It also controls naïve and memory T-cells and plays a key role in lymphopoiesis [196]. Impairments in IL7Rs are often reported in T-ALL diseases and they may depend on mutations occurring in exon 5 or exon 6 encoding for IL7Rα. The simultaneous role of IL7R as a target for NOTCH1, a generally mutated gene for T-ALL, also contributed to the pathogenesis [11, 197].

To date, several studies defined a direct relation between the signalling pathway of IL7R and the pathogenesis of T-ALL [194, 198]. One of the most recent investigations, based on a loss-of-function approach, formally demonstrated that the oncogenic programme of NOTCH1 and leukemia-initiating cells (LIC) activity were dependent on IL7R expression [199]. In this context, a fully human anti-IL7R antibody, which impaired IL-7/IL7R-mediated signalling, making T-ALL cells sensitive to chemotherapy, and promoting leukemia cell killing through a NK-mediated cytotoxicity, was generated [200]. In a subsequent study, a new anti-IL7R murine mAb, demonstrating its great efficacy through an ADCC-mediated mechanism against PDX T-ALL cells, was developed. Further in vivo studies proved the efficacy of the mAb on leukemic cell death both through ADCC-dependent and -independent mechanisms [201]. However, the presence of IL7R also in non-haematopoietic tissues makes this strategy still challenging; thus, further investigations are needed to confirm the efficacy and applicability in clinical practice.

### TCRβ

The TCRαβ is a heterodimer expressed on T cells, which interacts with molecules presented by the MHC. It contains two variable protein chains, namely TCRα and TCRβ combined with invariant CD3 molecules. TCRβ is composed by variable (V), diversity (D), joining (J), and constant (C) regions, while TCRα lacks the J-region. TCR diversity is strictly dependent by the recombination of the various domains resulting in specific T-cell clonality [202]. TCRβ-chain constant domains 1 and 2 (TRBC1 and TRBC2) are exclusively present on mature T cells while TRBC1 is also expressed in about 50% of TCR+ T-cell lymphomas. On these bases, a recent study focused on the development of third generation anti-TRBC1 CAR T cells, containing CD28 and OX40 co-stimulatory domains, to selectively recognize TRBC1+ cells in vitro and in vivo leukemia models. The selective killing of malignant T cells has been obtained using anti-Vβ8 and Vβ5 CAR-T cells, which preserve Vβ8- and Vβ5- normal T cells [202].

On these premises, an ongoing clinical study (NCT03590574) is investigating the safety and efficacy of a CAR-T cell treatment against TRBC1+ T cell malignancies.

Table 1 summarizes clinical trials involving CAR-T, mAbs or BTCE-based therapies for T-ALL treatment considering the aforementioned targets.

**Table 1:** CAR-T-, mAbs- or BTCE-based therapies currently under clinical investigation for T-ALL treatment

Target	Type	Intervention/treatment	Identifier	Stage	Investigators/responsible party
CD4	CAR-T	Anti-CD4 CAR-T cells for the treatment of r/r T-cell leukemia and lymphoma	NCT03829540	Phase 1	Indiana University
	CAR-T	Anti-CD4 CAR-T cells designed to treat leukemia and lymphoma expressing CD4 antigen	NCT04162340	Phase 1	iCell Gene Therapeutics
CD5	CAR-T	Donor-derived CD5 CAR-T cells in patients with r/r T-ALL pre-treated with chemotherapy	NCT05032599 NCT05487495	Phase 1	Beijing Boren Hospital
	CAR-T	CD5 chimeric receptor attached to CD28 from patient’s T cells or bone marrow transplant donor’s T cells for treatment of T-cell malignancies	NCT03081910	Phase 1	Baylor College of Medicine
	CAR-T	Anti-CD5 CAR-T cells for r/r T cell malignancies	NCT04594135	Phase 1	iCell Gene Therapeutics
CD7	CAR-T	Anti-CD7 CAR-T cells for the treatment of r/r acute leukemia	NCT04785833	Not applicable	PersonGen BioTherapeutics (Suzhou) Co., Ltd
	CAR-T	Anti-CD7 CAR-T cells for the treatment of r/r T-ALL or T-LBL	NCT04572308	Phase 1	Hebei Senlang Biotechnology Inc., Ltd
	CAR-T	Anti-CD7 CAR-T cells for r/r T-cell malignancies	NCT05290155	Phase 1	Shanghai Jiao Tong University School of Medicine
	CAR-T	T-cell injection targeting CD7 chimeric antigen receptor for T-cell malignancies	NCT04480788 NCT04004637	Phase 1	PersonGen BioTherapeutics (Suzhou) Co., Ltd
	CAR-T	Non-gene edited anti-CD7 CAR-T cells for patients with r/r T-ALL or leukemia	NCT04934774 NCT05212584	Phase 1	iCell Gene Therapeutics
	CAR-T	Anti-CD7 CAR-T cell in pediatric and young adult patients with r/r T-ALL or T-LBL	NCT04860817	Early Phase 1	920^th^ Hospital of Joint Logistics Support Force of People's Liberation Army of China
	CAR-T	Safety and clinical activity evaluations of allogeneic anti-CD7 CAR-T cells (ThisCART7) for patients with r/r T-cell malignancies	NCT05127135	Phase 1	Fundamenta Therapeutics, Ltd
	CAR-T	CD7 chimeric receptor attached with CD28 added to it to T cells for T-cell malignancies	NCT03690011	Phase 1	Baylor College of Medicine
	CAR-T	CAR-T cell therapy for patients with r/r T-ALL	NCT05043571	Phase 1	National University Hospital, Singapore
	CAR-T	Autologous CD7 CAR-T cells in therapy of high-risk acute T-cell leukemia/lymphoma	NCT04840875	Phase 1	Beijing Boren Hospital
	CAR-T	CD7 specific CAR gene-engineered T-cell for leukemia and lymphoma	NCT04033302	Phase 1	Shenzhen Geno-Immune Medical Institute
	CAR-T	Anti-CD7 allogeneic CAR-T cells safety and antitumor activity evaluations in patients with r/r T-ALL or LBL	NCT04984356 NCT05509855	Phase 1/2	Wugen, Inc
	CAR-T	Humanized CD7 CAR-T cells for r/r acute leukemia	NCT04762485	Phase 1/2	The First Affiliated Hospital of Soochow University
	CAR-T	Donor-derived CD7 CAR-T cells in patients with r/r T-cell leukemia/lymphoma; investigations on efficacy and safety	NCT04689659	Phase 2	Beijing Boren Hospital
CD38	mAb	Safety and efficacy evaluations of isatuximab SAR650984 in patients with T-ALL or T-LBL	NCT02999633	Phase 2	Sanofi
	mAb	Isatuximab combined with chemotherapy in pediatric patients with r/r T-ALL	NCT03860844	Phase 2	Sanofi
	mAb	Daratumumab in T-ALL young patients	NCT03384654	Phase 2	Janssen Research & Development, LLC
	mAb	Daratumumab-hyaluronidase for T-ALL patients with persistent MRD after treatment with chemotherapy	NCT05289687	Phase 2	Eastern Cooperative Oncology Group
	CAR-T	Humanized CD7 CAR-T cell therapy for r/r acute leukemia	NCT04762485	Phase 1/2	The First Affiliated Hospital of Soochow University
	BTCE	Safety and efficacy evaluation of XmAb18968 for patients with T-ALL, T-LBL and AML	NCT05038644	Phase 1	Medical College of Wisconsin
CD52	mAb	Investigation on efficacy and safety of alemtuzumab (Campath-1H) for patients with adult T-cell lymphoma	NCT00061048	Phase 2	National Cancer Institute, National Institutes of Health
	mAb	Association of alemtuzumab and fludarabine phosphate with low-dose total body irradiation before stem cell transplant for haematologic cancer	NCT00040846 NCT00118352	Phase 2	Fred Hutchinson Cancer Center
CD194	mAb	Mogamulizumab for r/r patients with T-cell malignancies	NCT04848064	Phase 1	Ohio State University Comprehensive Cancer Center

Data from clinicaltrials.gov

## Conclusions

**Fig. 3:**
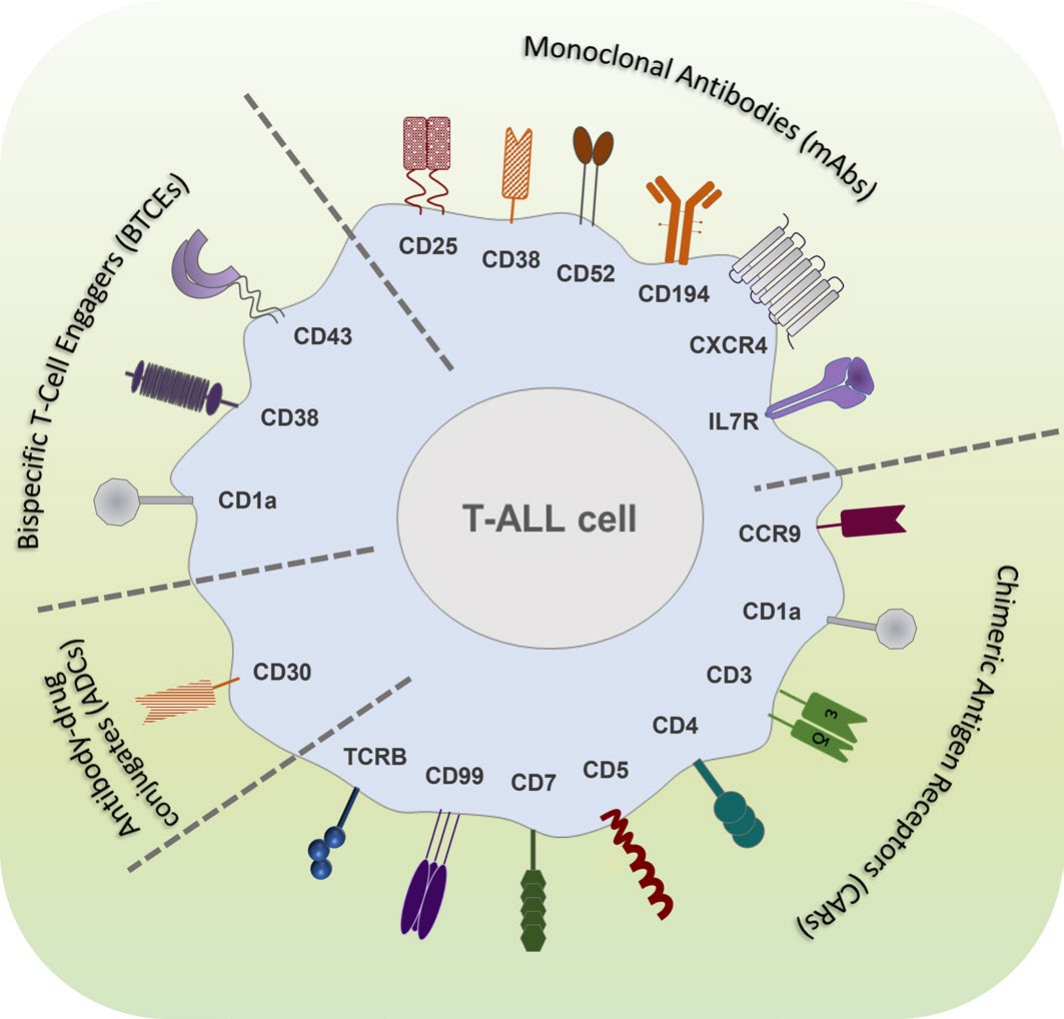
Schematic representation of immunotherapeutic approaches in T-ALL. Promising approaches based on monoclonal antibodies (mAbs), chimeric antigen receptor (CAR) T-cells and Bispecific T-cell engagers (BTCEs) to increase cure rates in T-ALL

T-ALL is a highly aggressive and lethal disease in relapsed/refractory patients. Although new molecular insights are driving the development of novel targeted therapy, the prognosis of T-ALL patients still remains poor. In this scenario, harnessing the immune system against leukemic T cells is a promising possibility on the therapeutic horizon for a durable response. Even if the shared expression of targets between normal and neoplastic T cells is the major obstacle to the development of effective strategies for T-ALL for life-threatening T-cell aplasia and severe opportunistic infections, new technologic options with CAR-T and BTCEs (Fig. 3) targeting highly selective T-ALL epitopes/antigens keep the promise to overcome these relevant challenges and improve the prognosis of this still orphan disease. In conclusion, immunotherapy is offering novel opportunities for T-ALL and the ongoing preclinical and clinical research will readily provide a more favorable clinical scenario in the next future.

## Data Availability

Not applicable.

## Abbreviations

T-ALL: T-cell acute lymphoblastic leukemia
BTCEs: Bispecific T-cell engagers
CAR: Chimeric antigen receptor
ALL: Acute lymphoblastic leukemia
WHO: World Health Organization
B-ALL: B-cell acute lymphoblastic leukemia
ETP-ALL: Early T-cell precursor lymphoblastic leukemia
CNS: Central nervous system
MTX: Methotrexate
MRD: Minimal residual dose
r/r: Relapsed/refracted
HCT: Haematopoietic cell transplantation
SCT: Stem cell transplantation
mAbs: Monoclonal antibodies
FcγRs: Fc regions to Fc gamma receptors
ADCC: Antibody-dependent cellular cytotoxicity
ADCP: Antibody-dependent cellular phagocytosis
CDC: Complement-dependent cytotoxicity
ADCs: Antibody–drug conjugates
AML: Acute myeloid leukemia
TCL: T-cell lymphoma
MHC: Major histocompatibility complex
TAAs: Tumor-associated antigen
scFv: Single chain variable fragments
IgG: Immunoglobulin G
FcRn: Neonatal Fc receptor
mBTCEs: Monovalent BTCEs
bBTCEs: Bivalent BTCEs
CRS: Cytokine release syndrome
ICANS: Immune effector cell-associated neurotoxicity syndrome
HSV-TK: Herpes simplex virus thymidine kinase
iCas9: Inducible cas9
PEBL: Protein expression blockers
ER: Endoplasmic reticulum
NK: Natural killer
ARDS: Acute respiratory distress syndrome
CRP: C-reactive protein
coT-ALL: Cortical T-ALL
TCR: T-cell receptor
FHV_H_: Fully human heavy-chain variable
PTCL: Peripheral T-cell lymphomas
TRAC: T-cell receptor alpha chain
NAD: Nicotinamide adenine dinucleotide
ATRA: All-trans retinoic acid
APL: Acute promyelocytic leukemia
ATL: Adult T-cell leukemia
PDXs: Patient-derived xenografts
HSPT70: Heat shock protein 70
Th2: Type 2 helper T-cells
IL-7: Interleukin-7
IL7Rα: Interleukin-7 α chain
LIC: Leukemia-initiating cells
